# Medical Association of Georgia

**Published:** 1887-05

**Authors:** 


### Society Reports.

### MEDICAL ASSOCIATION OF GEORGIA—THIRTY-EIGHTH ANNUAL SESSION.

ATLANTA, GA., April 20, 1887.

Dr. T. O. Powell, of Milledgeville, President, in the chair.

### FIRST DAY.

The Association met in the Senate Chamber at the Capitol and was called to order by the President at 10 o'clock.

The following members registered:

J. M. Hull, Augusta; W. B. Standifer, Blakely; J. L. Walker, Waycross; Robert B. Barron, Clinton; William Abram Love, Atlanta; T. S. Dekle, Thomasville; C. L. Sample, Cannoochee; E. W. Lane, Scarboro; N. P. Jelks, Hawkinsville; C. Hicks, Dublin; W. B. Cheatham, Dawson; J. L. Selman, Douglasville; P. L. Hilsman, Albany; E. C. Goodrich, Augusta; Eugene Foster, Augusta; William Perrin Nicolson, Atlanta; Mark H. O'Daniel, Milledgeville; C. P. Spier, Wadley; William A. O'Daniel, Bullards; Samuel C. Benedict, Athens; T. O. Powell, Milledgeville; A. G. Whitehead, Waynesboro; M. P. Deadwyler, Elberton; J. B. S. Holmes, Rome; K. C. Divine, Atlanta; J. L. Hamilton, Stone Mountain; W. Duncan, Savannah; A. A. Smith, Hawkinsville; T. L. Lallerstedt, Panola; James B. Baird, Atlanta; John C. Olmsted, Atlanta; W. S. Elkin, Atlanta; Virgil O. Hardon, Atlanta; T. R. Whitley, Douglasville; J. R. Henderson, Sun Hill; James F. Alexander, Atlanta; E. L. Connally, Atlanta; D. H. Howell, Atlanta; J. M. Boring, Atlanta; J. W. Duncan, Atlanta; F. M. Jordan, Hawkinsville; R. C. Word, Atlanta; W. A. Rosser, Bolingbroke; H. F. Scott, Atlanta; George C. Dugas, Augusta;

A. W. Calhoun, Atlanta; H. P. Cooper, Atlanta; James A. Gray, Atlanta; Wm. S. Armstrong, Atlanta; Paul Faver, Fayetteville; John Gerdine, Athens; John G. Earnest, Atlanta; T. J. Word, Atlanta; W. G. England, Cedartown; W. D. Bizzell, Atlanta; W. L. Sterling, Atlanta; J. S. Todd, Atlanta; G. G. Roy, Atlanta; J. P. Logan, Atlanta; J. McF. Gaston, Atlanta; J. M. Kelley, Griffin; J. M. Head, Zebulon; Robert Battey, Rome; Charles F. Benson, Atlanta; J. M. McDowell, Barnesville; L. H. Jones, Clarkston; J. F. Anderson, Cornucopia; P. R. Cortelyou, Marietta; Arch. Avary, Atlanta; W. F. Yarbrough, Oxford; E. G. Lind, Atlanta; Wm. H. Leyden, Atlanta; A. G. North, McDonough; R. L. Tye, McDonough; T. D. McKown, Jonesboro; A. G. Hobbs, Atlanta; E. Van Goitsnoven, Atlanta; T. E. Collier, Atlanta; Thomas D. Love, Atlanta; H. V. M. Miller, Atlanta; J. H. Heard, Walden; B. L. Joiner, Andersonville; A. B. Ashworth, Atlanta; R. O. Cotter, Macon; R. O. Engram, Montezuma; G. W. Mulligan, Washington; T. S. Fortson, Washington; T. M. Holmes, Rome; W. O'Daniel, Bullards; I. H. Goss, Fort Lamar; W. P. Ponder, Forsyth; R. J. Nunn, Savannah; L. J. Hardman, Harmony Grove; J. B. Pendergrass, Jefferson; E. C. Goodrich, Augusta; A. W. Griggs, West Point; W. F. Westmoreland, Atlanta; J. A. Beasley, West Point; J. W. Bailey, Gainesville; F. R. Calhoun, Euharlee; J. G. Greene, Acworth; John A. Callaway, Milledgeville; W. B. Parks, Atlanta; J. H. Duggan, Stephensville; V. H. Taliaferro, Atlanta; R. P. Sorrell, Harmony Grove; A. C. Davidson, Sharon; J. H. Green, Decatur; T. Harry Huzza, Atlanta; J. L. Barge, Osanda; Dewell Gann, Bolton; C. C. Green, Atlanta; M. C. Duncan; J. F. Cole, Carrollton; R. H. Taylor, Griffin; F. J. Arbeely, Atlanta; E. R. Anthony, Griffin; T. J. Charlton, Jr., Savannah; F. M. Ridley, LaGrange; J. W. Clements, Subligna; E. H. Richardson, Cedartown; Thos. S. Powell, Atlanta.

Prayer was offered by Rev. Henry Clay Morrison, pastor of the First Methodist Church of Atlanta.

The address of welcome was delivered by Dr. J. F. Alexander, of Atlanta. Dr. Alexander said it afforded him great pleasure to welcome the doctors to the city. The annual convention of the physicians of the State did great good by affording opportunity for an interchange of thoughts and opinions, and also gave much pleasure in a social way. Dr. Alexander, while welcoming the doctors to the city, said he thought that as Georgia's citizens they had cause to be proud of Atlanta—a city of morality, religion and advancement. He spoke of the healthfulness of the city, the splendid climate, the mineral springs adjacent, and especially did he speak of the "famous Salt Spring, the wonder of this section, now being improved and beautified by enterprising citizens."

DR. FOSTER RESPONDS.

Dr. Eugene Foster, of Augusta, responded to the address of welcome.

Dr. Foster said that as Paris was France, so it seemed Atlanta was about to be considered all of Georgia. Everything comes to Atlanta. About seventy-five years ago, he had an inclination to fight Atlanta, but he had learned to love the city. He wished every city in Georgia was like Atlanta, and that the grand old city of Augusta had Atlanta's public spirit, and enterprise, and local confidence. When Atlanta had reached a population of 5,000, a friend of his was asked what foundation the place rested on and what her future promised. The reply was: "God Almighty only knows, but since the place has got to be a town of 5,000, I see no reason why it shouldn't be as big as London." Atlanta always had open arms for the Medical Association, always wanted the Association to meet within her borders and he always felt at home here, When Dr. Calhoun, in passing through the crowd, had said. "Make yourselves at home," an apt reply was made by one who said, "Why shouldn't we? It is our town." You may turn the keys over to us, said Foster; we will go where we please, do what we please, and when we get through we will turn the keys over to you again.

The Secretary then announced the programme for the session; following this announcement the President read his annual address. His subject was

HEREDITARY ENVIRONMENT.

He said there is no question of such vital import to the human race and none so much neglected as heredity and environment so far as they relate to this race. In this address I must be content to speak only of some of the most formidable inherited tendencies to disease, and the induced or acquired morbid habits that are readily transmitted, and which do not appear in the descendants in a stereotyped form, but are manifested in an endless variety of morbid forms. Every tendency or quality of the physical and mental condition of man, such as will, intelligence, sentiment, passion, etc., of the healthy and morbid alike, is capable of more or less direct transmission. Hence heredity and environment are very important factors in the formation of the character of a people, mentally, physically and morally. These features are fixed in a people as in a family. Heredity can be traced directly through some of the most formidable diseases to which we are subject; and when both parents have defective organisms, it is a physical certainty that their peculiarities will be manifested in their offspring in some form. Ribot says: "Heredity is that biological law by which all beings endowed with life tend to repeat themselves in their descendants." The masses look to the medical profession for the enunciation of the laws of life and health, and it is certainly incumbent upon us to inculcate hygienic truths, and, as far as possible, impress upon the minds of the masses the danger of disregarding that biological law of heredity and environment. It behooves the profession not only to give their hearty sympathy, but to be co-workers also in every effort that tends to promote the health and improvement of the human race. But this duty does not devolve upon the medical profession alone, and the neglect of heredity and environment cannot be laid solely at their door. The responsibility rests upon all professions alike, inasmuch as a violation of the inexorable law of nature and the legitimate results of its violation are destructive to society, to the powers of a government, and to the physical and psychological powers alike of the human race. It is evident to all who have given any attention to heredity that the outcome of violating natural laws is to increase the mental anxiety, the burdens, the disease, the suffering, the depravity and crime of a people. Our civilized and Christian communities and governments foster nurseries for breeding, developing and perpetuating these morbid tendencies. It is a blot upon our civilization, and, alas, be it said, upon our Christian intelligence. The laws of health, religion, obedience and civilization are inseparable. They go "hand-in-hand." Our civilization is due to religious influences. If we could get the true etiology of much of our depravity and crime, it would point directly to heredity and bad environment as the predisposing and exciting factors. In my judgment, we will not be justified in hoping for an elimination, or even an abatement of these inherited morbid tendencies so long as they are fostered by society and a mistaken benevolence.

But our susceptibilities to them will continue to increase in each succeeding generation in the same ratio that we disregard the functions that resist disease and neglect the proper means for neutralizing and eliminating the poison that lurks within our constitution, whether there by transmission, or induced, or acquired by our own acts through the influence of our surroundings. The sooner we recognize the fact that the native, mental, physical and moral endowments are inherited just as readily as the general appearance in form and features, the better for our race; and better still, when we recognize the truth that maladies and abnormal conditions are, to a large extent, preventable. Hence the necessity of knowing our antecedent history, and that our natural tendencies to disease should be held in check by avoiding all external surroundings that would be likely to damage the physical and mental integrity, considering at all times and under all circumstances the family defects. No family should hesitate or fail to give to their family physician, as far as possible, a full and complete history of their antecedents, direct and remote, their tendencies to health and disease, their tastes, habits, and idiosyncrasies, but should confer with the physician freely as to the natural tendencies of their children, and as to what sort of mental training and environments they should have, and even in regard to the business callings of life. The transmission of physiological morbid processes is seen every day, on every side and in every community; And it should be remembered that this law is operative alike upon both the mental and physical powers. It has been my duty for years to care for many of this unfortunate class, and my observation, and the records of the institution over which I preside will show to our shame that a majority of these maladies point back to heredity as the most potent cause; and they show further that it is not necessary, by any means, that the defects of the parent should be fully developed in order to transmit to the children tendencies to morbid conditions. I dare say that all ailment, and the histories of all cases in similar institutions, if they could be correctly obtained, would bear me out in these statements. It would not be difficult to produce instances of families, once possessed of great mental and physical powers and good morals, who have married into defective families, and continued to do so for pecuniary and other considerations, until they have degenerated, physically, mentally and morally, becoming a burden to themselves and to society, and in some cases these families have faded out entirely, without giving a thought to heredity, or to the inevitable consequences of the violation of that biological law. The strong stand by almost without an effort to correct or check these great evils, or to prevent the perpetuation of such deplorable conditions, when so much might be accomplished by proper training and education—the only forces through which help can be had. Is not this a sad commentary on our civilization and Christian intelligence? There was a bill introduced in the last Legislature looking to the education of our youth in the laws of life and the functions of living beings. What became of it? It died for the want of sufficient support. I cannot say why it was not supported, but the probabilities are that the vital importance of this measure to the human race was not fully appreciated. If we neglect to educate the masses so that they will understand and appreciate the laws of health and the natural principles which underlie human development, degeneration will follow. It is not presumed that mankind in general, and especially the young, when they are so succeptible to all sorts of bad influences, will guard against danger unless they apprehend that danger exists; but when the danger is known, to avoid it is natural. Why is it that we see the people who dwell near the track of the cyclone insuring their property and digging pits? The answer is easy. They understand and appreciate the danger to life and property from these storms, and they do all in their power to guard their lives and property against future injury. Is it not reasonable to suppose that we would have the same forethought and observe the same caution if we knew that we were susceptible to any particular disease; if we knew that there was a sleeping magazine deeply and securely fixed within our constitution simply needing a match, the slightest exciting cause, to produce a fearful and destructive explosion? With such a knowledge, would we not cultivate the healthy tendencies and develop stronger and more vigorous constitutions, so as to be better qualified to resist the attacks of destructive influences; and if the morbid tendencies are not cultivated, they will gradually, in many cases, become atrophied, and, with proper marriages and healthy surroundings, will ultimately fade out. Many of us are living to-day by accident. Some of our ancestors had perhaps consumptive diathesis, and by fortuitous circumstances married into vigorous and healthy families, free from all unhealthy taint, and hence we are living.

Levy says: "To be born of healthy and strong parents is to have a good chance of longevity." If the laws of biology and the effects of the violation of the physiological laws were properly understood, is it not reasonable that they would be regarded more than they are now? The only hope is in the proper mental training and education at home and in our schools. If this should fail, would it not be just, wise and humane, and would not the dictates of humanity demand that there should be a board of health in every county, composed of the best, the most cultured and conscientious physicians, whose duty it should be to give a certificate, so far as relates to the mental and physical condition of parties, before they could obtain marriage license? I fully appreciate the difficulties and the apparent impracticability of such a law, and allude to it simply for the sake of humanity. I know that the hue and cry would be that the liberties and the rights of the people were being taken from them; but so far as their rights are concerned, do not such marriages not only take away the liberty of their posterity, but do they not surely entail upon them deformity, suffering, disease, depravity, crime and death? Is it not true that a large number that spring from such marriages ultimately drift into the penitentiaries; that many die in early life from consumption and alcoholism; that others die on the gallows and by suicide; many get into alms-houses; others drift into various humane and charitable institutions? If this class can be deprived of liberty by a jury of twelve men, why could not the defectives, with equal justice and humanity, be deprived of the right to intermarry, and leave such a legacy to their posterity and to society? Is it surprising that like should generate or produce like? If not, then the nature of heredity for the good of the human race should engage our most earnest and active attention. We have wholesome laws in regard to some diseases, by which the liberties of the people are restricted to prevent their spread, because it would be hurtful to the public health. For instance, yellow fever, small.pox, etc.; individuals and communities are quarantined, and in the case of small-pox, they are required, or forced, if necessary, to submit to vaccination. These diseases are not half so formidable and destructive, or hurtful to the public health, or the productive powers of a government, or the harmony of society, as some hereditary diseases or acquired morbid conditions which are readily transmitted in various morbid forms, such as alcoholism, syphilitic taint and the like, nor does it cost millions to care for them. It is evident that the masses do not consider or appreciate predisposing causes of disease, or the potentialities of the future in regard to them. We are frequently asked if we would advise against the marriage of children whose parents were the subjects of hereditary taint, or acquired morbid habits that are transmitted. Only under certain circumstances would we advise against it. Investigation, before advising, should relate to the nature and cause of the morbid tendencies, and the amount of predisposition to a diseased diathesis, and whether inherited or acquired, and if manifested in the parents in any way prior to the conception or birth of the children; and in this connection it should be remembered that the normal and morbid, in their physical and mental natures, are governed by the same law. All of the indications of each case should be thoroughly considered. Accidental causes that occur after the birth of children should not be regarded as predisposing causes, nor should simply imbecility. No one having a remote or direct tendency to a consumptive diathesis should marry into a family with the same diathesis, or a neurotic or insane diathesis, for insanity is frequently a symptom of consumption. They keep pace one with the other, and there is a close relation between the two. Nor should they marry into families of alcoholic diathesis, or families of syphilitic taint. In other words, defective organisms should not intermarry, but they and their descendants should marry into families of vigorous, healthy constitutions, free from all defects, at the same time cultivating the normal and holding in check the morbid by good environments. In my judgment, if all the endowments were well considered in marriage and pursued methodically, the general results would be for the improvement of the human race. The phenomena of reversional heredity, or ativism, occasionally occurs in the human race in apparently healthy families, having children of defective organisms. We sometimes see this illustrated in color alone. I know a tall, bright mulatto man who married a bright mulatto, and they have nine children, all mulattoes, save two. One of these is a blended color, while the other is as black as black can be; but he is the image of his father in every particular, shape, form, height, feet, limbs, carriage and voice, having all of the characteristics of his father, and to see them together would be to know that the tall, black man was the son of the mulatto. His grandmother was also a very bright mulatto, but his grandfather was a short, stout, very black Negro, and this man simply inherited the color of his grandfather. In regard to ativism, or universal heredity in the lower order of animals, the stock-raisers appreciate it and understand it. They generally call it "breeding back," a very good name. In the human race, when this law is indicated in a tendency to be disobedient, cruel or depraved on the part of one of the children of a good moral family, the child manifesting these tendencies generally receives the same routine training and is influenced by the same surroundings at home and school as the other children of different tendencies; hence this condition grows with his growth, and the family is frequently very much surprised at his conduct. As soon as he gets from under the family influence, he comes in contact with bad environments that are fostered by society, and he goes rapidly to ruin, leaving, perhaps, a blot upon the otherwise bright pages of the family record. If the natural tendencies of this child had been properly considered, and his mental training and surroundings made favorable, it is probable that the trouble would have been overcome. Such children are usually dubbed "the black sheep" of the family. It is through the influence of this law and bad environment that many strange, sad and unexpected things occur in some of the very best families. Many other illustrations might be given in various forms of morbid conditions and tendencies, but the law of reversional heredity is so well established by endless instances that it is not necessary to name them.

The laws of heredity are fully appreciated in their influence upon the lower order of animals in determining strength, speed, symmetry, endurance, docility, etc., and those engaged in breeding fine stock carefully scan the animal from head to foot, and make the most diligent inquiry as to the antecedent history, pedigree and indeed all the qualities before they will select it for breeding purposes. If it is so important to recognize this law in obtaining such qualities as we may desire in the lower order of animals, why is it not of equal importance in the human race when we know the same law is operative alike in this race? I was forcibly impressed with the attention given to the law of heredity in rearing fine horses, while in Kentucky. On one occasion I had the pleasure of visiting a noted stock farm. There I found a highly intelligent gentleman, a scientific man, engaged in rearing fine horses. They were noted for their beauty, speed and endurance, and when I went into his barn I saw there the pictures of the original horse and some of his descendants. The first was one of the most ill-shaped, abnormal-looking things I ever beheld. He had one quality which was desired to be retained and improved, namely, speed. He was regarded as fast in his day, and with proper surroundings and judicious crossing it was hoped that the defects in symmetry might be eradicated. As the pictures went around on the wall, from one generation to another, the improvement was marked, step by step, until the defects in symmetry had been entirely eliminated, and the speed of the family had been wonderfully improved. It is a matter of unspeakable shame that we have not yet learned to value the human animal as men value the horse or the cow, or even the pig. Children are bred, taught and developed without reference to natural tendencies or the grand result which should be the aim of all training. Of many of our race it may be said, "They were not brought up; they just growed." I might say here that the manner in which our children are taught at home and in our schools is frequently productive of much physical and mental suffering. Parents and teachers, over-anxious to push their children forward, stimulate them to longer hours, and crowd them with far more studies than is conducive to their psychical and physical health. These should be developed in harmony one with the other; and it should be remembered that no two children are any more alike mentally and physically, than they are in appearances, features, etc. Their natural tendency should be well considered, and each child's mental training and surroundings should be determined with an eye to their deficiencies and their needs. I apprehend that this is one of the principal troubles. Teachers should not be overcrowded with pupils so that they may have opportunities of studying the peculiarities or natural tendencies of each child. No educator can do justice to a child without such a knowledge. I could illustrate by pointing to many cases that have come under my observation, minds and bodies broken down by these hurtful processes. Parents and teachers were so blinded by their anxiety to see the pupils progress rapidly that they could not see that such great and continuous mental effort in the early formative stage of life was more than even an adult brain with the same tendencies could stand. All educational systems should be viewed from a physiological and sanitary standpoint. Moral education should be an important feature of home and school-training. A failure to educate children to obey is fraught with much suffering to both children and parents, and this particular element of successful training should claim our most earnest attention. Defective education increases the depravity and disease of a people; all educational systems that disregard the natural tendencies and moral training are defective. From what germ came our depravity, suffering and death? From disobedience on the part of Adam and Eve The same law is still in force; it is the inexorable law of God. If children learn to obey from their youth, it will be a fixed principle of their mature life, and their will-power will be sufficiently strong to come in contact with the business callings of life. Such an education enables them the better to conform to all the laws of health. Self-control is elevating, socially and morally, and it is absolutely necessary to health and happiness. The action of the brain is more or less under control of will-power. It controls the depraved tendencies, keeping them under subjection, and leads the mind constantly into normal channels which give healthy mental action. If obedience to law is not observed in youth, the volition will be too weak to resist the depraved tendencies, passions and appetites, and utter ruin will, in all probability, be the result when the untrained come in contact with the world. It is of profound importance to parents and educators that the will-power of children be strictly observed. There is a commendable interest being manifested in the humane care, treatment and reformation of the criminal. Some of the most philanthropic people have organized a National Prison Congress, and are giving much time and thought to the best means of caring for and reforming this unfortunate class. While I know and appreciate the fact that this congress originated in the heart of a noble, philanthropic,

Christian gentleman, and that it has accomplished good, and will accomplish more, yet I believe that if half of the commendable zeal manifested for the welfare and reformation of these unfortunates, after they get into trouble, was manifested in the correction of their hereditary tendencies, and in securing proper attention to hygienic surroundings in youth and healthy marriages, there would be much less depravity and crime and far less use for Sing-Sing prisons or convict camps, nor would there be so great a demand for alms-houses, inebriate asylums and other charitable and humane institutions.

And would not this be the course of true benevolence? I am satisfied if this congress could get at the true etiology of the crime of every criminal, much of it would be due to heredity and bad environment. Much of this trouble is susceptible of mitigation or even entire elimination; but we will not be satisfied in looking for such reformation in adult life. It must be done in youth.

Proper education of the masses is the only force by which we can hope to attain such an end. The science of health must be taught at home and in our schools. Maudsley says: "Knowledge of ourselves, of our body and the various functions of its organs, and the effects of external objects, would be a most effectic antidote for most of the evils which afflict mankind." It is not only the duty of the profession, but of society, to ascertain the most important factors in the causation of our evils, predisposing and exciting, and enforce such wholesome laws and hygienic surroundings as will check them. Pardon me for saying just here that, in my judgment, Atlanta, the largest city in the State, and one of the most enterprising and public-spirited in the United States, has made a noble and manly effort to remove from her midst one of the most potent factors in the causation of all of our evils. There is a problem not far in the future that will greatly concern the Southern States; and it will not be a matter of little moment. I refer to the care of the criminal and afflicted of the colored race. Unless there is a radical change in their habits and surroundings we may expect great trouble in the future. The habits and environments of the women are very much like those of the men; and when both parents have bad habits, or defeetive organizations, it is almost a certainty that the children of such parents will be depraved or defective. Like will beget like. "Whatsoever a man soweth that shall he also reap." I confidently expect the race to be to us what the defective foreign immigration is to many of the Northern, Western and Eastern States. Up to 1867 there was no class of people that enjoyed better physical and mental health than the Negroes of Georgia. They were then most entirely exempt from insanity and consumption. They are now very susceptible to both, and the rapid increase of these diseases is alarming, when we know that the genuine Negro in slavery had no inherited predisposition to such a diathesis. Consumption and insanity keep pace one with the other. This fact is well illustrated in the Negro. His race had one tendency that was perhaps inherited, and that was a disposition to steal, and I do not know that they were much to blame for it in that day, as they felt that what their masters had was made by slave labor, and if the laborer could take it without being detected, it was all right and no wrong was done. The successful thief was regarded by his associates as bright and shrewd. The crime had no tendency to lower the thief in the estimation of his race, but rather to elevate. Their condition now, in that particular, reminds me of an anecdote I once heard of a politician seeking an office at the hands of the Legislature. I will not say whether this occurred in Milledgeville or Atlanta. The politician was, of course, passing around, shaking hands with all the members and expressing his pleasure on seeing them. While making his rounds, he meets a young member and immediately exclaims: "Why, I am so glad to see you; how are you? Your father is a warm friend of mine, and I am so much attached to him. How is your father!" "He is dead." "Why, I am sorry to hear it. When did he die?" "Some time ago."

After this conversation the office-seeker proceeded to tell what he wanted to do in the service of the dear people and passed on. Later in the canvass he came around again to this same young member and was very glad to see him. "Your father is my special friend," says he. "How is he?" "He is still dead, sir," was the reply."

Judging from the convict camps, I apprehend the Negroes still have that tendency. As I have said, there is danger that the colored race will be to us, without a radical change, what the defective immigration is to many of the Northern, Western and Eastern States. While we have been comparatively exempt from this class, we cannot say how long we will be. It is well known that the United States is the asylum for the defective classes of Europe. They have immigrated to the United States, and here they have intermarried, and continued to generate that defective and depraved class until the situation in many of those States has become alarming. It takes millions upon millions to care for them, and those States are endeavoring, through their State Medical Associations, to get Congress to prevent their being made a garbage box for Europe. "While it is said that this foreign-born immigration only constitutes one-eighth of the population, they furnish one-third of the paupers, one-third of the criminals and one-third of the insane." At the last meeting of the State Medical Society of Wisconsin, held in Madison in June last, the following resolutions with reference to the immigration of the defective classes were adopted:

"WAEREAS, It is known that a large number of foreigners be longing to the defective classes, such as paupers, criminals, the insane, deaf mutes, blind idiots and lepers are annually shipped to this country from other nations; that insanity, pauperism and crime are increasing rapidly in this country; that the chief cause of this increase is due to the large number of defectives found among the foreign born; and

"WHEREAS, The present national law is not sufficiently potent to guard against this indiscriminate immigration; and whereas, the individual States and Territories cannot act independently: therefore be it

"*Resolved*, That the President of this Society be, and he is hereby empowered to appoint at this session a committee of three of its members to act in the name of the Wisconsin State Medical Society in presenting a memorial to the next Legislature, with urgent request that our Legislature take immediate steps to place the matter properly before Congress, which body alone must take final action; and

"*Resolved*, That a copy of these resolutions be presented to each of our United States Senators, to each of our Congressmen and to the President of each State Medical Society in the United States."

I feel that we ought to give to our Wisconsin Medical Society our hearty co-operation, and I hope this Association will take such action as may be deemed advisable in the matter, inasmuch as they have called upon the Medical Association of Georgia to aid them.

To give you some idea of the ground for apprehension so far as it relates to the insane, I will name the total population and the total insane, the ratio of insane to total population, the native population, native insane, and the ratio to native insane, the foreign population, foreign insane and ratio of foreign insane to foreign population, in several of the States; and I doubt not the same ratio will hold good in the pauper and criminal classes.

New York has a total population of five millions eighty-two thousand eight hundred and seventy-one. Total insane, fourteen thousand one hundred and eleven, or one to every three hundred and sixty-two. The native population is three millions eight hundred and seventy-one thousand four hundred and ninety-two, and the native insane is seven thousand seven hundred and ninety, or one to every four hundred and ninety-eight. The foreign population is one million two hundred and eleven thousand and three hundred and seventy-nine; the foreign insane is six thousand three hundred and twenty-one, or one to every one hundred and ninety-one. California has a total population of eight hundred and sixty-four thousand six hundred and ninety-four. Total insane, two thousand five hundred and three, or one to every three hundred and forty-five. The native population is five hundred and seventy-one thousand eight hundred and twenty; the native insane is eight hundred and eighty-five, or one to every six hundred and forty-six. The foreign population is two hundred and ninety-two thousand eight hundred and seventy-four; the foreign insane is one thousand six hundred and eighteen, or one to every one hundred and nineteen.

Wisconsin has a total population of one million three hundred and fifteen thousand four hundred and ninety-seven; total insane is two thousand five hundred and twenty-six, or one to every five hundred and twenty-one. The native population is nine hundred and ten thousand and seventy-two; the native insane is one thousand and fifty, or one to every eight hundred and sixty-six. The foreign population is four hundred and five thousand four hundred and twenty-five; the foreign insane is one thousand four hundred and seventy-six, or one to every two hundred and seventy-four.

These figures were obtained from the census of the United States for 1880. In view of facts like these, we cannot wonder at the great concern the matter is giving the people of these States, and at the action of their State medical associations in regard to it. Indeed, it is not very extravagant to say that there is ground for fear that the time may come when there will not be a sufficient number of healthy organisms in this country to restrain or control the defective. With the Negroes in the South more and more tending to disease and depravity, and the defective foreign-born population in the North and West, the future is anything but bright.

Now, gentlemen, in conclusion I will simply refer to some of the needs of the State without going into detail, inasmuch as everything looking to the care of suffering humanity is a legitimate subject for the consideration of this Association.

1. There is no enterprise that commends itself more to our profound sympathies than a school for the feeble-minded or idiotic children of our State. Its great utility and value would soon be seen under wise and patient training. There are many cases that are susceptible of great improvement, and some cases that might be qualified to sustain themselves. It is wrong to associate them with the insane, for it is hurtful to them and to the insane, and also to the State in an economical view.

2. There should also be established a hospital for the care and treatment of the insane convicts. The wards of ordinary asylums for the insane are not proper places for the custody and treatment of the insane convict. It is impossible to afford the necessary protection against their escape, and it is a crying shame to associate the insane with them; the moral effect is very detrimental to other patients. With such a hospital connected with some one of the convict camps, and with proper protection against their escape, there would be much less feigning insanity among them.

3. One of the great necessities of the State is a house of correction for the depraved children. The young, with depraved tendencies that are, perhaps, inherited, should not be placed in the penitentiaries with surroundings that foster and develop these tendencies. When with proper care and environments, such as moral education and training, they might be reformed and made self-sustaining and useful citizens.

This class of children are frequently sent to the insane asylum, a very improper place for them, for they come in contact with the delusions, hallucinations, the perverted ideas and actions of the insane, which is damaging to them, and it is impossible to give them the necessary moral education and employment that is absolutely necessary for their reformation.

Dr. Eugene Foster introduced the following resolution:

*Resolved*, That in view of the great value of the President's address to the public, as well as to the medical profession, that copies thereof be furnished to the press with requests for publication.

Which was adopted.

Dr. William Perrin Nicolson offered a resolution providing that a committee of one from each congressional district be appointed to consider and report on the recommendations made in the President's address. The resolution was adopted.

Dr. A. A. Smith offered a resolution inviting members of the press, the clergy and visiting physicians to seats with the Association. The resolution was agreed to.

### REPORTS OF COMMITTEES.

Reports of committees were next in order.

Dr. H. V. M. Miller, of the Committee on Expert Testimony, stated that a bill had been prepared and presented to the Legislature, but that body had failed to pass it. He had no idea the bill would pass, but as there would be a summer session, asked that the committee be continued, which was agreed to.

Dr. Miller made also a report for the Committee on Medical Legislation. He stated that a bill had been prepared and submitted to the Legislature to facilitate the study of anatomy, and that the bill was not satisfactory, but the question was not what the doctors wanted, but what they could get. It was better than no bill at all, and he had some reason to believe it would pass. He did not know that it would receive the signature of the Governor. A bill with the same object was once vetoed by the Governor after it had passed.

Dr. Wm. Abram Love, of the Committee on State Medical Directory, made a partial report and asked for further time, which was granted.

Dr. Love introduced a resolution indorsing the American Medical Association and pledging the sympathy of the State Association in the efforts to make the International Medical Congress a success.

The resolution was promptly carried.

The Secretary then read a telegram from Dr. Dunglison, Treasurer of the American Medical Association, asking aid for the International Congress.

Thereupon Dr. Miller moved to reconsider Dr. Love's resolution, saying he had not understood it fully. He said the Association would be called on for contributions it could not afford to make.

Dr. Battey spoke, saying the expenses of the Congress were enormous; that the government's appropriation was inadequate, and he did not think it advisable to pass windy resolutions unless the State Association was willing to go down deeply into its pockets and help out.

The resolution was reconsidered, and, on motion, was laid on the table to be called up to-morrow.

The Association then adjourned to meet at 3 o'clock at the Union Passenger Depot to go on an excursion to Salt Springs. The train reached the village of Salt Springs at 4 o'clock, and found waiting there the narrow-gauge train ready to take the excursionists to the spring. There was very little delay, and in a few minutes the doctors and other members of the party were freely imbibing the famous water.

The pavilion at the springs is a very handsome one, while the bath-house is a perfect model. After a half-hour's stay at the spring, drinking water, admiring the scenery and enjoying the delightful retreat in the fullest measure, the Association was photographed by a representative of Parke, Davis & Co.

On their return to the village, the Association spent an hour or so inspecting the magnificent new hotel now nearing completion. The hotel is one of the most beautiful buildings to be found anywhere. It is situated in a beautiful grove, forty acres of which will be made into a lovely park. The hotel contains two hundred and forty-five rooms and cost $165,000.

Mr. Marsh and his associates gave the Association a magnificent banquet; at the conclusion of the banquet, Dr. Eugene Foster was called on to return the thanks of the Association for the hospitalities extended by Mr. Marsh. He responded in a pleasant manner.

---

SECOND DAY.

The Association was called to order at 10 o'clock by the President.

Dr. S. L. Ledbetter was introduced as fraternal delegate from the Alabama State Medical Association.

Dr. W. D. Bizzell, of the Committee on Re-organization, made a report, which was ordered printed. It provides a new constitution and by-laws, and will come up for discussion at the next annual session.

Dr. Jerome Cochran, of Alabama, was introduced to the Association. He was received with applause, and addressed the Association, explaining how the success of the Alabama Association had been secured. He said a great deal was due to the county organizations.

He said that if he was not greatly mistaken, there would be no such thing as irregular practice in Alabama in ten years.

Dr. Nunn read a bill which had been prepared, making it a misdemeanor for any physician to disclose any professional secrets obtained from patients in examinations made in the practice of medicine or surgery. The bill was referred to the Committee on Legislation.

### THE ORATOR'S ADDRESS.

At half-past twelve the orator's address was delivered by Dr. William A. O'Daniel, of Bullards. The address dwelt with the duties of doctors and with the peculiar sphere occupied by the physician, and was an exceedingly able and interesting discourse.

During the afternoon the sections were called and reports were made and papers read by the following members: Dr. R. J. Nunn, of the first district, on Gynæcology; Dr. P. S. Hilsman, of the second district, on Surgery; Dr. R. O. Ingram, of the third district, on Surgery; Dr. A. A. Smith, of the third district, on Gynæcology; Dr. A. W. Griggs, of the fourth district, on Surgery; Dr. J. McF. Gaston, of the fifth district, on Surgery; Dr. Virgil O. Hardon, of the fifth district, on Gynæcology; Dr. W. O'Daniel, of the sixth district, on Practice; Dr. T. M. Holmes, of the seventh district, on Practice. The other districts are yet to be heard from.

### THIRD DAY.

The Association was called to order at 10 o'clock by the President.

Dr. Eugene Foster read a paper on the therapeutic value of alcoholic liquors. It was a review of a paper read by Dr. Joseph P. Logan, of Atlanta, before the last meeting of the Association, on the use and abuse of alcoholic liquors, which was published in THE JOURNAL.

Dr. Foster took the opposite view, and argued that alcoholic liquors were valuable agents in the cure of many diseases, and that the

LOCAL OPTION LAW WAS WRONG

in denying to physicians the right to use them, thus robbing the profession of one of its chief remedies. He said that great indeed must be the urgency of the case where the people of a State or country dared take upon themselves the right to invade the domain of scientific medicine and say, "Here are certain articles which, although we find in your materia medica, you shall not prescribe, even if you propose to use them solely as medicines." Such a thing, Dr. Foster said, could not be justified among people who had not run mad with fanaticism.

Dr. Foster said that he had long been a temperance man, and that he had observed the baneful effects of alcoholism and was always ready to join in any reasonable measure to suppress intemperance, but he could not indorse every means resorted to. The temple of medicine had been invaded by the crusaders because a few learned and honored physicians had expressed grave doubts as to the value of liquors as drugs.

Dr. Foster took up the points made by Dr. Logan and answered them one by one. He quoted extensively from textbooks to show the value of alcohol as a remedial agent and to show that liquors had a well-defined place in the list of medicines. He said he saw before him men whose lives he believed had been saved by the use of alcohol as a remedy.

It was an able paper and elicited the closest attention from every one.

Dr. Logan replied briefly, stating in substance that he had, of course, no idea of attempting, at present, to cover the ground occupied by Dr. Foster. He still had confidence in the positions taken in his paper of last year, and if "one fact was worth a thousand theories," he had several facts which he thought would successfully meet the large mass of theory contained in Dr. Foster's paper. As his introduction of the consideration of the use and abuse of alcoholic liquors in medicine before the Association had been characterized as fanatical, he had submitted the paper to the criticism of two of the most distinguished medical men in the United States, Dr. Henry F. Campbell, an ex-president of the American Medical Association, and an eminent physician and surgeon of Georgia, and Dr. N. S. Davis, the originator of that Association, the editor of its journal, President of the International Medical Congress, and the equal of any medical man in America. He read some extracts from the lengthy letter of Dr. Campbell, endorsing fully the propriety of the discussion of the subject before the Association, and referring to some misrepresentations of his views as antagonistic to Dr. Logan's paper, stating that he was not only impressed at the time of the reading of the paper with the propriety of the discussion, but also with its ability and fairness, and that while long observation and habit of thought had placed him among those who attach a high value to alcohol in certain conditions of disease, it was only as a remedy and not a beverage that it could have his approval, classing it among the deadly poisons, as strychnine, arsenic, atropia or opium, if improperly used. He also commented upon the loose manner in which alcohol was prescribed by physicians sometimes, and asserted that the medical man who would inconsiderately prescribe a tonic dose of brandy or whisky to a patient for the "stomach's sake before meals" would almost surely make him a drunkard. "After all this," he remarked, "decided comment upon my part, defending and commending your address, agreeing with you *in the main*, and only mildly but conscientiously differing with you on the point of excluding alcoholic stimuli, I was greatly disappointed and mortified to find in the daily paper next day, that Dr. Campbell had answered Dr. Logan adversely, and had recommended alcoholic beverages as valuable for the sick." He desired his letter to be used as a vindication of both Dr. Logan and himself from false position and concluded by stating that "I have very carefully read the address as published in THE ATLANTA MEDICAL AND SURGICAL JOURNAL. It again impresses me as a fair, just and careful consideration of the subject of alcoholic stimulants as related to the present spirit of the age. You give high authority and good prestige for the views you advocate, and as your sincere and long-time friend, I will say you have no just reason to feel the least solicitude in regard to the acceptance it will receive at the hands of the profession as an inquiry into the effects of alcohol on the system, and as a discussion looking to more accurate methods of its administration as a medicinal agent in such conditions of the system as may require its application." Dr. Logan then read the following brief letter from Dr. N. S. Davis in reply to a request for his criticism upon the paper referred to, containing not only its full endorsement, but furnishing some important statistics bearing upon the question at issue, and tending to show the progress which is being made in its elucidation:

CHICAGO, ILL., March 19, 1887,

65 Randolph street.

*J. P. Logan, M. D.*—DEAR DOCTOR: Your letter and paper were received to-day. The position you take in regard to the effects of alcoholic liquors upon the human system and human society, and the ability of the medical profession to dispense with their use entirely without injury to the sick, is correct and susceptible of full practical demonstration. Probably few general practitioners of medicine have treated a larger number of patients of all classes than I have during the last thirty years, and yet during that whole period I have not prescribed for internal use a *pint* of alcoholic liquors, either fermented or distilled. As a single specimen of results, since October 1st, 1886, there have been admitted into the clinical wards of the Mercy Hospital, under the care of myself and of Dr. N. S. Davis, jr., more than thirty cases of well-marked typhoid fever, some of them unusually severe. They were freely accessible to the *clinical class* from the college each week. Not a spoonful of alcoholic liquor was used in their treatment, and yet all of them recovered. Can you find any better results under the *administration* of *alcoholics?*

Yours truly, N. S. DAVIS.

Dr. Logan then presented some statistics bearing upon the question of the treatment of medical and surgical disease without alcohol (though, as he stated, never having committed himself absolutely to its exclusion) from the London Temperance Hospital, under the professional charge of such eminent physicians and surgeons, equal to any in London, as A. Pierce Gould, F. R. C. S., James Edmunds and Robert Lee, the former, upon invitation of the late Dr. Austin Flint, having sent a paper to the Surgical Section of the American Medical Association at its annual meeting in Washington in 1884, entitled "A Year's Surgery at the London Temperance Hospital," which was received and published in the journal of the Association September 13th, 1884, Vol. 3d, page 288, the latter two being members of the International Medical Congress in London, 1881, the "London Temperance Hopital," being fully recognized as one of the general hospitals of London. The reports from this hospital, from which extracts were read by Dr. Logan, exhibited the following facts: The reports framed in regard to the surgical cases during the first 1½ years show that 52 surgical cases had been in the wards of the hospital, besides the usual minor operations among the out-patients, (all of which latter did well), and in no one of these cases had alcohol been prescribed. Among these 52 cases no death occurred, eight of the number requiring surgical operations, including amputation of a portion of the hand, resection of the hip-joint, resection of the elbow-joint, removal of necrosed bone from the spine of the scapula and various tumors.

In the treatment of 401 surgical cases afterwards up to April, 1884, treated without alcohol, in which there were 103 surgical operations, there were only three deaths. There were five deaths among the cases not requiring operations, making a total of eight deaths among the 401 surgical cases, and in no one of these, from their almost necessarily fatal character, can it be suggested that alcohol would have prevented the issue. In the Thirteenth Annual Report of the same hospital ,presented May, 1886, the object of the institution is stated to be the alleviation and cure of disease by means which the highest science of the day approves, and at the same time to protect the sick from a form of temptation peculiarly subtle and dangerous. Any wish to sacrifice scientific methods to philanthropic impulses is disclaimed, but it is believed that all good men and women will rejoice if it can be shown that the disuse of alcohol as a medicine is not detrimental to those who are subjected to medical and surgical treatment. The medical staff have the power to prescribe alcohol as a drug if they consider a trial of its use needful, but have only so regarded it in one case during the year, and in that it was used without any apparent beneficial result. The in-patients received during the official year were 624, and as 48 were under treatment May 1st, 1885, the total number treated was 672; the number under treatment May 1st, 1886, being 53, the deaths were 41, giving a rate of mortality of 6.7 per cent. From the opening of the hospital, October 6, 1873, to April 30, 1886, twelve years and seven months, the in-patients were 3,486, of whom 1,968 were cured, 1,272 relieved and 183 died, a percentage of 5.2. It is further stated that undeniable and numerous signs are testifying to the diminished faith of intelligent practitioners in the supposed therapeutic virtues of alcohol, and that the average amount of alcohol used in hospitals and infirmaries has decreased and is still decreasing with evident advantage to the patients. Even in those special diseases, for the cure of which alcohol was believed to be almost indispensable, such as acute pneumonia, typhoid fever, small-pox, etc., it is now found by those who are bold enough to dispense with its use that results are obtainable tending to disprove the assumption, not only of its necessity, but of its value. From another source, Dr. Logan stated that it was said that the results of treatment in this hospital were 4½ per cent. more favorable than in any hospital in London, and at least, it cannot be disputed that the statistics are equally favorable. In conclusion, Dr. Logan said, in reply to Dr. Foster's call upon him for a substitute, that while he could furnish a long list, such as ammonia in its various preparations, heat, the ethers, camphor, aromatic essential oils, oil of turpentine, counter-irritants, digitalis, belladonna, strychnia, coffee and tea, yet he was under no obligation to do this, unless it could be demonstrated that *alcohol is a necessity* in the treatment of disease, which is a manifest impossibility, as it could not be *proved* that it has ever saved a single life.

After the conclusion of Dr. Logan's remarks, on motion of Dr. R. J. Nunn, of Savannah, Dr. Foster's paper was ordered published in THE ATLANTA MEDICAL AND SURGICAL JOURNAL.

Dr. W. F. Westmoreland presented a case he termed the double-headed boy. He said the boy had at the lower extremity of the spinal column what seemed to be an extra head. The outlines of the nose, mouth and eyes were well marked. The growth was covered, except what seemed to be the face, with long curls, not unlike the hair on the head. The boy was eleven years old. He had been exhibited in all parts of the United States as the double-headed boy, and up to within a few months before he was brought to this city had enjoyed good health. About six or eight weeks before he was brought here, the extra head began to give him pain and very soon began to suppurate. In a few weeks the boy showed well marked symptoms of septicæmia, and when presented to Dr. Westmoreland he advised the removal of the mass. The parents objected at first, saying that they had consulted the best surgeons in the country and they had advised against any operation, saying that it would surely produce death whenever the mass was removed. They reluctantly gave their consent, however, and the operation was made. Dr. Westmoreland stated that the cocyx and a portion of the sacrum were involved in the tumor or head and had to be removed. Dissection revealed very well-marked cranial bones in the mass with a membrane similar to the dura-mater, but there was no brain substance in the sac. He presented the boy and the tumor. The boy has entirely recovered from the operation.

Dr. R. C. Word read a most interesting paper on "The Duality of the Brain with a Theory for Mind-reading and Slate-writing."

Mind-reading, according to Dr. Word's theory, is a phase of mesmerism. The mesmerized man is in what he styles an electro-negative or passive condition. There are probably 20 per cent. of mankind who are constitutionally electro-negative. Such are very easily mesmerized, and perhaps there are 20 per cent. of people who are partially electro-negative, and who, by practice and perseverance, may be brought under the mesmeric influence. Females are more susceptible than males.

The more frequently a person is mesmerized the more sensitive and susceptible he becomes, so that he may even throw himself into that state and voluntarily become electro-negative to any one with whom he is in *rapport;* the word *rapport* meaning contact or in position where a nerve or magnetic current may flow between the parties. It is possible for a number of persons to be in *rapport* at the same time.

The mind-reader is an electro-negative subject, who by practice has become exceedingly sensitive to impressions, so that he is liable to be influenced by any one with whom he comes in contact, especially if the person whose mind he seeks to read will concentrate his thoughts upon any particular object by which he becomes more positive, and the more easily influences or dominates the other who is mesmerized, or at least passive or recipient in his nervous relation to the person who is positive.

Dr. Word said there were many grades or phases of the mesmeric state. Some were in a hypnotic or unconscious condition; some were partially so, and others were fully awake and yet impressible and subject to the mesmeric influence. He expressed the position that one side of the brain might be electro-negative to the other side, so that a kind of intercourse might exist between them, the one side conversing with the other side, so to speak. This, he said, really occurred with the slate-writer, or so-called spirit-writer. Thoughts, incidents and latent memories from one side of the brain are written automatically and seem to the other, or conscious side, to come from an outside or third party.

The paper was listened to with interest by every one.

After the reading of Dr. Word's paper, Dr. Love called from the table his resolution expressing regret over the differences existing in the American Medical Association and sympathy with the aims of the International Congress.

Dr. Love said that in 1884 the American Medical Association instructed its delegates to the International Medical Congress, at Copenhagen, to invite the congress to meet in Washington in 1887. After the invitation was accepted, the delegates usurped the duties of the American Medical Association, and, organizing themselves into a committee, arranged for the International Congress. At subsequent meetings the conduct of the delegates was revised, in so far as it related to the appointments for the congress, and hard feelings were engendered. Dr. Love's resolution was an endorsement of the efforts being made by the American Medical Association to make the congress a success.

Dr. Gaston spoke in support of the resolution.

Dr. Baird thought it best to take no action.

After a short discussion the resolution was agreed to.

The following report of the auditing committee was made and adopted:

Your Committee on Auditing Accounts and Books of the Secretary and Treasurer beg leave to repoit as follows:

The books of the Treasurer are well and neatly kept, and show a balance of $111.89 in the treasury at the opening of this session, a condition of financial healthfulness which our treasury has but seldom ever known. Certainly we owe many thanks to our polite, attentive and efficient Treasurer.

We find that the Secretary is faithfully and efficiently discharging the duties of his office, reflecting honor upon the Association as well as himself. In addition to the laborious and valuable services of his office, he has added $125.25 to the treasury by industrious work in securing advertisements inserted in book of Transactions.

Drs. Goodrich and Gray are entitled to the thanks of this Association for their work in promoting the interests of the Association. Respectfully submitted.

EUGENE FOSTER, Chairman.

The President announced the following as delegates to the American Medical Association:

A. G. Whitehead, Eugene Foster, R. J. Nunn, H. V. M. Miller, Robert Battey, W. F. Westmoreland, J. P. Logan, W. O. Daniel, G. W. Mulligan, E. H. Richardson, James A. Gray, E. C. Goodrich, William Duncan, C. L. Sample, T. S. Dekle, P. S. Hillsman, H. F. Scott, B. R. Doster, T. S. Powell, W. D. Bizzell, A. A. Smith, John Gerdine, S. B. Hawkins, M. H. O'Daniel, J. S. Todd, R. C. Word, I. L. Harris, J. A. Beasley, A. W. Griggs, J. F. Anderson, J. A. Callaway, E. L. Connally, W. F. Holt, C. H. Hall, K. P. Moore, T. M. Holmes, M.P. Deadwyler, J. W. Bailey, A. W. Calhoun, J. McF. Gaston and T. S. Hopkins.

The following voluntary papers were submitted and referred to the publication committee:

"Importance of the Early Treatment of Insanity. Avantages of Hospitals over Home Treatment." By Dr. Mark H. O'Daniel, of Milledgeville.

"A report of eleven cases of Sudden Illness, six of which proved Rapidly Fatal." By Dr. John A. Callaway, of Milledgeville.

"Measles." By Dr. L. H. Jones, of Milledgeville.

"Hydrastis Canadensis in Uterine Troubles." By Dr. R. B. Barron, of Clinton.

"Morbid Reflex Uterine Neuroses." By Dr. Wm. Abram Love, of Atlanta.

"Mechanical Treatment of Vomiting in Pregnancy." By. Dr. J. W. Flanders, of Wrightsville.

"Successful Treatment of Bright's Disease of the Kidneys with Double Chloride of Gold and Skim Milk." By Dr. J. W. Flanders, of Wrightsville.

"Practice Long Time Ago." By Dr. Henry Gaither, of Oxford.

"Treatment of Corneal Ulcers by Actual Cautery." By Dr. R. O. Cotter, of Macon.

"A Case of Utero Vesico Vaginal Fistula." By Dr. V. H. Taliaferro, of Atlanta.

"Hereditary Glaucoma." By Dr. A. W. Calhoun, of Atlanta.

The following officers were elected:

President—Dr. A. G. Whitehead, of Waynesboro.

Vice-Presidents—Dr. A. A. Smith, of Hawkinsville, and Dr. John Gerdine, of Athens.

Secretary—Dr. James A. Gray, of Atlanta.

Treasurer—Dr. E. C. Goodrich, of Augusta.

Censor—Dr. M. H. O'Daniel, of Milledgeville.

Orator—Dr. J. M. Hull, of Augusta.

The Association then adjourned to meet in Rome on the 3rd Wednesday in April, 1888.

